# Differential adaptability between reference strains and clinical isolates of *Pseudomonas aeruginosa* into the lung epithelium intracellular lifestyle

**DOI:** 10.1080/21505594.2020.1787034

**Published:** 2020-07-22

**Authors:** Maria Del Mar Cendra, Eduard Torrents

**Affiliations:** a Bacterial Infections and Antimicrobial Therapies Group, Institute for Bioengineering of Catalonia (IBEC), the Barcelona Institute of Science and Technology, Barcelona, Spain; b Microbiology Section, Department of Genetics, Microbiology and Statistics, Faculty of Biology, University of Barcelona, Barcelona, Spain

**Keywords:** *Pseudomonas aeruginosa*, intracellular persistence, lung, epithelial cells, clinical isolates, host-pathogen interactions, intracellular lifestyle, chronic infections, cystic fibrosis, ribonucleotide reductase

## Abstract

Intracellular invasion is an advantageous mechanism used by pathogens to evade host defense and antimicrobial therapy. In patients, the intracellular microbial lifestyle can lead to infection persistence and recurrence, thus worsening outcomes. Lung infections caused by *Pseudomonas aeruginosa*, especially in cystic fibrosis (CF) patients, are often aggravated by intracellular invasion and persistence of the pathogen. Proliferation of the infectious species relies on a continuous deoxyribonucleotide (dNTP) supply, for which the ribonucleotide reductase enzyme (RNR) is the unique provider. The large genome plasticity of *P. aeruginosa* and its ability to rapidly adapt to different environments are challenges for studying the pathophysiology associated with this type of infection.

Using different reference strains and clinical isolates of *P. aeruginosa* independently combined with alveolar (A549) and bronchial (16HBE14o- and CF-CFBE41o-) epithelial cells, we analyzed host–pathogen interactions and intracellular bacterial persistence with the aim of determining a cell type-directed infection promoted by the *P. aeruginosa* strains. The oscillations in cellular toxicity and oxygen consumption promoted by the intracellular persistence of the strains were also analyzed among the different infectious lung models. Significantly, we identified class II RNR as the enzyme that supplies dNTPs to intracellular *P. aeruginosa*. This discovery could contribute to the development of RNR-targeted strategies against the chronicity occurring in this type of lung infection.

Overall our study demonstrates that the choice of bacterial strain is critical to properly study the type of infectious process with relevant translational outcomes.

## Introduction

Laboratory reference strains fail to mimic *in vivo* bacterial infections [1]. Their high genome plasticity drives bacteria to rapidly adapt to the habitat where they grow [2]. Differences in sequence or genome size can be found in the same bacterial species depending on whether it has been isolated *in situ* from an infection or whether it comes from a laboratory collection after being passaged for decades under particular laboratory conditions [3]. Hence, the phenotype of a laboratory strain will rarely recreate the *in vivo* infectious environment [1].

*Pseudomonas aeruginosa* is a Gram-negative ubiquitous bacterium that behaves as a nosocomial pathogen in humans. The large genome plasticity of *P. aeruginosa* confers it the ability to live in a wide range of environments, from a free-planktonic lifestyle to living in pathogenic and disease environments [4]. The ability of *P. aeruginosa* to grow in very diverse environments and oxygen atmospheres is due in part to the different classes of ribonucleotide reductase (RNR) encoded in its genome: class Ia (*nrdAB*), II (*nrdJab*) and III (*nrdDG*) RNRs [5]. RNR is responsible for providing the deoxyribonucleotides (dNTPs) required for DNA synthesis and repair; hence, it is an essential enzyme in all living cells. The requirements for generating the radical needed to perform catalysis differ among RNR classes. Class I RNR depends on oxygen to generate a tyrosyl radical; therefore, it is only active under aerobic conditions. Class II RNR uses 5ʹ-deoxyadenosylcobalamin to generate a cysteinyl radical and is oxygen independent. Finally, class III RNR requires strict anaerobic conditions to generate an oxygen-sensitive glycyl radical using S-adenosylmethionine [6]. *P. aeruginosa* can balance the expression of the three known RNR classes to allow its growth in extremely distinct environments, which is an ability not shared among bacteria and makes *Pseudomonas* a versatile and tenacious bacterium [5,6].

Under appropriate conditions, *P. aeruginosa* can cause an extensive variety of infections, such as bacterial keratitis, endocarditis, encephalocarditis, otitis, pneumonia, wound infections, and even septicemia. The lungs are specifically affected by this pathogen. In the lungs, *Pseudomonas* is able to cause both acute and chronic pneumonia [7]. Chronicity is caused by the capacity of *P. aeruginosa* to grow in highly protective and resistant communities called biofilms, as well as to promote cellular invasion as a mechanism to evade the host immune system [8,9]. People affected with life-threatening cystic fibrosis (CF) are particularly worried by this pathogen since it is responsible for the majority of morbidity and mortality associated with this disease [7–9].

A continuous supply of dNTPs is indispensable during an infectious process. Marked oxygen deprivation in tissues is a signal of disease and infection [10]. In this study, we used the invasive PAO1 and cytotoxic PA14 laboratory strains together with different clinical CF isolates of *P. aeruginosa* to evaluate the differences in the intracellular persistence, and modulation of the expression of the RNR classes, during infection depending on the natural environmental background of the strain. The adenocarcinomic alveolar A549 cell line together with the bronchial 16HBE14o- and its CF-derived cell line CFBE41o- were the epithelial cell lines used in this study. CFBE41o- cells encode a stable ∆F508 mutation in the *CFTR* gene to promote the CF phenotype. Furthermore, since the three lung epithelial cell lines are genetically and phenotypically different, for instance, high hypoxia levels have been found in the tumor-immortalized cell line A549 [11], while increases in reactive oxygen species production have been detected in CF-derived CFBE41o- cells [12], cell type-directed infection by a specific *P. aeruginosa* strain of a particular lung epithelial cell line was examined.

Our work demonstrates the differential cellular invasion, intracellular persistence, and oxygen consumption patterns among the reference and clinical isolates of *P. aeruginosa* studied toward the different pulmonary epithelial cells. Thus, confirming different *P. aeruginosa* host-pathogen behaviors depending on the natural genetic background of the infectious bacteria. Significantly, by revealing a clear and marked transition from class Ia RNR during planktonic life to class II RNR expression during the intracellular persistence of the bacterium among the different lung epithelial cell lines, this study identifies class II RNR as the dNTP supplier required for the *P. aeruginosa* intracellular lifestyle.

## Materials and methods

### Bacterial strains and growing conditions

In this study, we used the *Pseudomonas aeruginosa* PAO1 (CECT 4122/ATCC 15,692) and PA14 [13] reference laboratory wild-type strains and different cystic fibrosis isolates, PAET1, PAET2, and PAET4, which were isolated from recurrent infections of three different chronic-infected patients [14]. The three different clinical isolates differed in the nature of infection they were isolated from: while PAET1 and PAET2 strains were isolated from patients that were not previously colonized, the PAET4 strain was isolated from a CF-patient that suffered from recurrent infections. In this sense, PAET1 and PAET2 could be treated as early isolates whereas PAET4 as a late *P. aeruginosa* clinical isolate. The isogenic *nrdJ* and *nrdD* mutants in the PAO1 strain were also used [15]. Overnight (O/N; ~16 h) cultures of *P. aeruginosa* were routinely grown in Luria-Bertani medium (LB; Scharlab) at 37ºC with vigorous shaking. LB-agar (Scharlab) was used to count the respective bacterial colony forming units (CFU) of the *P. aeruginosa* strains.

### *Complementation of* P. aeruginosa *PAO1 Δ*nrdJ *and PAO1 Δ*nrdD *strains*

To complement *P. aeruginosa* PAO1 Δ*nrdJ* strain, the encoding region of *nrdJab* gene from *P. aeruginosa* PAO1 was cloned into the *Bam*HI sites of the pUCP20 T vector generating the pETS218 vector. Otherwise, for *P. aeruginosa* PAO1 Δ*nrdD* complementation, we used the pETS197 plasmid [14], which expresses the encoding region of PAO1’s *nrdD* gene. Each plasmid was electroporated inside the respective PAO1 Δ*nrdJ* and PAO1 Δ*nrdD* strain. Carbenicillin at 300 ug/mL was used for plasmid maintenance.

### Lung epithelial cells

Three different epithelial cell lines of the human airway were used in this study: adenocarcinomic alveolar A549 (ATCC® CCL-185), bronchial 16HBE14o- and cystic fibrosis bronchial CFBE41o-. CFBE41o- epithelial cells are homozygous negative for the ∆F508 mutation of the *CFTR* gene [16]. For each set of experiments, a new vial was thawed, and cells were passaged no more than 10 times. Cells were cultured in T-75 tissue culture flasks (Thermo Fisher Scientific) and grown in Dulbecco’s Modified Eagle Medium: Nutrient mixture F12 (DMEM/F12; Thermo Fisher Scientific). Media were supplemented with 10% (v/v) decomplemented fetal bovine serum (dFBS), 100 Units/mL penicillin and 100 Units/mL streptomycin (Thermo Fisher Scientific). Cells were passaged and maintained in a humidified incubator at 37ºC and 5% (v/v) CO2 (ICOmed, Memmert).

### P. aeruginosa *adhesion, invasion and intracellular persistence in lung epithelial cells*

A549, 16HBE14o-, and CFBE41o- cells were seeded into 24-well culture plates (SPL Life Sciences) at ~7.5x10^4^ cells/well and incubated for 48 h prior to O/N incubation with antibiotic-free DMEM medium. Incubation without antibiotics was performed to ensure that possible traces of antibiotics that could interfere with the experiments were cleared. Infection with the different *P. aeruginosa* strains was performed at a multiplicity of infection (MOI) of 100 in antibiotic-free DMEM for 3 h. To determine bacterial adhesion (adhesion and invasion), cell monolayers were gently washed three times with warm 1x phosphate-buffered saline (PBS; pH 7.4) and lysed with 200 µl of PBS containing saponin (0.1% w/v; saponin buffer) for 15 min. To quantify the intracellular invasion of the different *P. aeruginosa* strains, we used the gentamicin survival assay as previously described [17]. Briefly, after 3 h of infection, monolayers were washed with warm 1X PBS and then incubated with DMEM containing gentamicin 200 µg/mL (gentamicin solution) for 90 min to kill extracellular *P. aeruginosa*. After incubation with the gentamicin solution, cells were washed with warm 1X PBS and subsequently lysed with the same volume of saponin buffer used for the adhesion evaluation. Serial dilutions were plated onto agar plates, and colony-forming units (CFU) were enumerated after incubation at 37ºC O/N. The invasion rate of each *P. aeruginosa* strain per cell was calculated as a percentage of the invaded CFU relative to that adhered.

To determine the intracellular growth of the different *P. aeruginosa* strains inside the different lung epithelial cells, monolayers were infected at a MOI of 1 for 3 h and then incubated with a gentamicin solution. Bacterial invasion was evaluated after 1.5 h, 3 h, 4.5 h, 6 h, and 21 h of incubation at 37ºC. When the complemented *P. aeruginosa* PAO1 Δ*nrdJ+*pETS218 and PAO1 Δ*nrdD+* pETS197 were used, Cb 300 µg/mL was added to the culture medium to ensure the maintenance of the plasmid. The slope of intracellular survival of each *P. aeruginosa* strain after 21 h of Gm treatment was calculated with the GraphPad Prism 8.0 software.

### Protein electrophoresis and staining

The *P. aeruginosa* protein expression patterns of the different strains were determined by sodium dodecyl sulfate–polyacrylamide (SDS-PAGE) gel electrophoresis. Briefly, 3 μg of total protein from the PAO1, PA14, PAET1, PAET2, and PAET4 strains grown under planktonic conditions for 3 and 24 h was loaded on a 10% SDS-PAGE gel. Protein quantification of each crude extract was determined by the Bradford assay (Bio-Rad). After electrophoresis, the gels were stained using PageBlue^TM^ Staining Solution (Thermo Fisher Scientific) following the manufacturer’s instructions.

### *Western blot of Nrd proteins from* P. aeruginosa

Western blotting was performed to analyze the differences in the intracellular expression of the NrdA, NrdJ, and NrdD proteins among the *P. aeruginosa* strains. Confluent monolayers of A549, 16HBE14o-, and CFBE41o- were infected with the *P. aeruginosa* reference PAO1 and PA14 strains in addition to the CF isolates PAET1, PAET2, and PAET4 strains at a MOI = 100 for 3 h. At the time point, each extracellular fraction was centrifuged, and bacterial pellets were lysed with BugBuster extraction reagent (Novagen) following the manufacturer’s instructions. Otherwise, two different bacterial fractions were used to evaluate intracellular Nrd protein expression: one fraction was taken after 3 h of infection and the other fraction was taken after 24 h of intracellular persistence. To do so, infected monolayers were washed with warm 1X PBS and incubated at 37ºC with a gentamicin solution for 90 min or for 21 h to kill extracellular bacteria. After incubation, cells were washed with 1X PBS, and monolayers were lysed with BugBuster lysis solution.

Five micrograms of total protein were separated by SDS-PAGE and electrotransferred onto polyvinylidene difluoride membranes. Detection of NrdA, NrdD, and NrdJ by Western blot analysis was performed as we previously described [5] using anti-NrdA, anti-NrdD, and anti-NrdJ (Agrisera, Sweden) antibodies at a 1:1000 dilution. Detection of primary antibodies was performed using a donkey antirabbit horseradish peroxidase-conjugated secondary antibody (Bio-Rad) at a 1:50,000 dilution. Immunodetection was performed using the Amersham^TM^ ECL^TM^ Prime Western blotting reagent (GE Healthcare) according to the manufacturer’s instructions, and proteins were visualized using the ImageQuant^TM^ LAS4000 mini system (GE Healthcare). Unspecific bands were used as loading controls. A pilot Western blot experiment using a crude protein extract of the lung monolayers failed to detect the NrdA homologue in human RRM1 (~90 kDa), thus confirming the specificity of the anti-NrdA antibody against the bacterial protein. NrdA (~107.1 kDa), NrdD (~76.1 kDa), and NrdJ (~82.7 kDa) protein bands were selected according to the molecular weight given by the antibodies binding to purified NrdA, NrdD, and NrdJ proteins from our laboratory stock (Figure S1A). Unspecific protein bands from each Nrd protein immunoblot were used as a loading control (Figure S1B). Protein band analysis was performed using ImageQuant^TM^ LAS4000 software, and the average number of pixels of each Nrd protein band volume was used as relative to the levels of protein expression. To calculate the induction of each Nrd protein after 24 h of intracellular persistence, the average number of pixels of each protein band was normalized by the average of pixels determined in the corresponding unspecific band shown in Figure S1B.

### *Quantitative Real-Time PCR (qRT-PCR) of* nrdA, nrdJ *and* nrdD *genes*

Intracellular expression of *P. aeruginosa nrd* genes was assessed by doing a qRT-PCR with samples taken after 3 and 24 h of PAO1, PA14, PAET1, PAET2, and PAET4 intracellular persistence inside A549 monolayers. The infection conditions were the same as those used for the western blot analysis. At each time point, infected monolayers were washed with 1X PBS and lysed with saponin buffer. Samples were treated with RNAlater (ThermoFisher Scientific), to preserve the integrity of the RNA. RNA was isolated using the GeneJET RNA Purification kit (ThermoFisher Scientific) following the manufacturer’s instructions. RNAs were treated with DNase I (Ambion) to ensure that samples were clear of DNA traces.

Total RNA samples were adjusted to 1 μg and retrotranscribed using the Maxima reverse transcriptase (ThermoFisher Scientific) and random hexamer primers (ThermoFisher Scientific).

qRT-PCR of *nrdA, nrdD, nrdJ* genes, in the different infectious conditions and time points was done with 1 μl of cDNA per reaction, using PowerUP Sybr Green Master Mix (ThermoFisher Scientific) and specific primers of *P. aeruginosa*’s *nrd* genes [18]. The glyceraldehyde 3-phosphate gene (*gapA*) was used as an internal control. qRT-PCR was performed in a StepOnePlus real-time PCR system (Applied Biosystems), and the 2^−ΔΔCT^ method was used to analyze the data and determine each intracellular gene induction between time points.

### Fluorescence microscopy and cellular hypoxia determination

A549, 16HBE14o-, and CFBE41o- cells were independently seeded into a Millicell® EZ SLIDE with eight wells (Merck Millipore) and left until confluence. For this experiment, phenol red-free DMEM/F12 (ThermoFisher Scientific) was used to avoid autofluorescence of the medium when visualized under a microscope. Monolayers were infected with the different *P. aeruginosa* strains for 3 h at a MOI = 100. After infection, the cells were washed 3 times with warm 1X PBS and then incubated with a gentamicin solution supplemented with the hypoxia probe dye (Organogenix) for 30 min (~3 h time point postinfection) and 21 h (24 h time point postinfection) independently. This probe allows the detection of environments with low oxygen levels since its phosphorescence is quenched by oxygen; therefore, its signal increases in response to a low oxygen content (red fluorescence). The hypoxia probe was used following the manufacturer’s instructions (Organogenix).

Stained monolayers were visualized under a Nikon inverted fluorescence microscope ECLIPSE Ti-S/L100 (Nikon) coupled with a DS-Qi2 Nikon camera (Nikon) to detect changes in the intracellular oxygen content depending on the *P. aeruginosa* strain and/or lung cell type. Analysis of the images obtained was performed using ImageJ FIJI software.

### *A549, 16HBE14o- and CFBE41o- viability after 24 h of* P. aeruginosa *intracellular persistence*

Lung cell viability was assessed in a 96-well microtiter plate after 3 h of infection with the *P. aeruginosa* strains PAO1, PA14, PAET1, PAET2, and PAET4 at a MOI = 100 followed by 21 h of incubation with gentamicin solution by using the PrestoBlue^TM^ Cell Viability Reagent (Invitrogen). Cells incubated with dimethyl sulfoxide (DMSO) for 21 h were used as a positive control for toxicity. PrestoBlue^TM^ is quickly reduced by metabolically active cells; hence, higher absorbance values correlate to greater metabolic activity. The reagent was used according the manufacturer’s instructions. Briefly, at the previously mentioned time point, 10 µl of PrestoBlue^TM^ was added to each well, and the plate was subsequently incubated for 1 h at 37ºC. After incubation, absorbance was read at λ = 570 using a microplate reader (Infinite M200 Microplate reader, Tecan). The given values were corrected by the absorbance at λ = 600 values as recommended by the manufacturer.

### Statistical analysis

The adhesion *vs* invasion values of *P. aeruginosa* were compared using an unpaired *t*-test. Otherwise, differences in bacterial intracellular CFUs/monolayer over time, monolayer toxicity among the different *Pseudomonas* infections and differences between pixel intensities were analyzed by using one-way ANOVA with Dunnett’s multiple comparison test with GraphPad Prism 8.0 software.

## Results

### *Specialization of CF clinical isolates of* P. aeruginosa *to interact with alveolar and bronchial epithelial cell monolayers*

**Figure 1.**
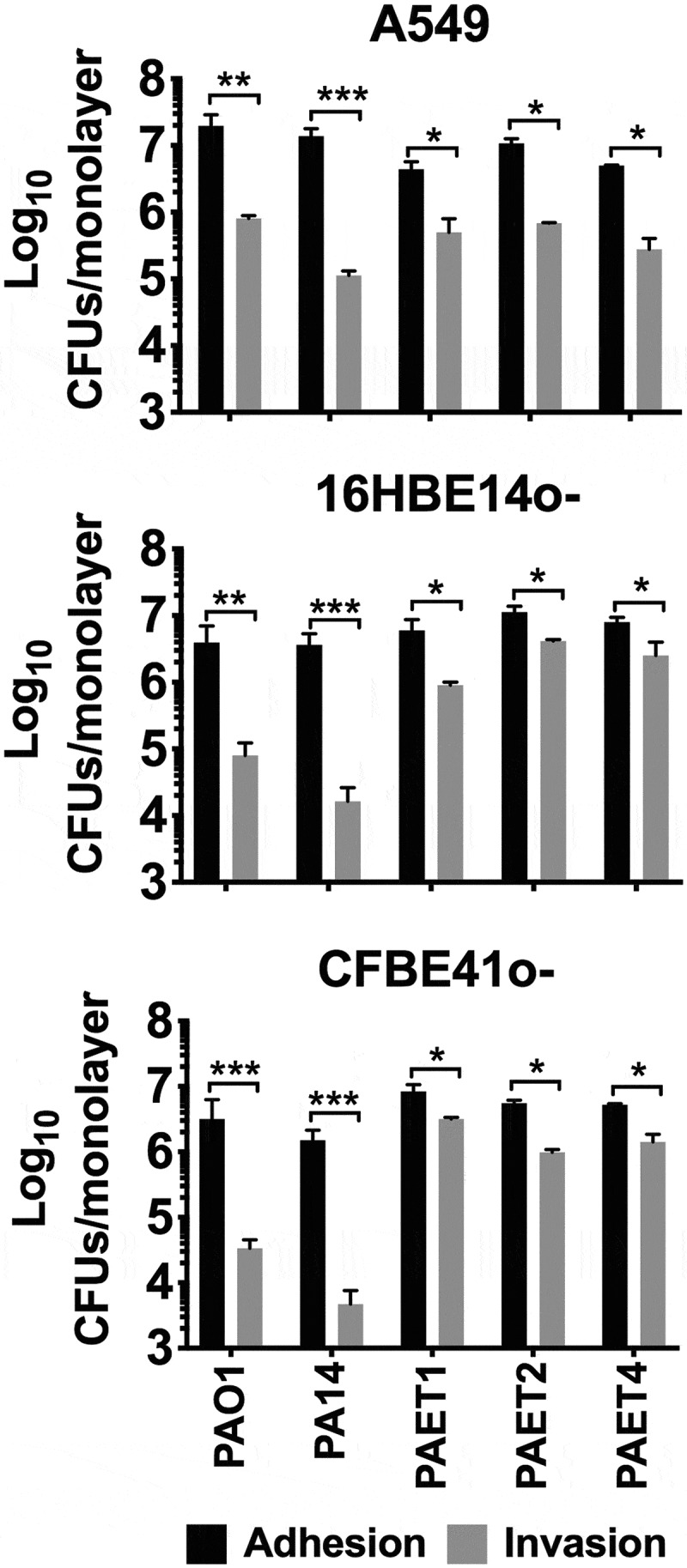
Interactions of *P. aeruginosa’*s with alveolar and bronchial lung epithelial cells. The adhesion and invasion values of *P. aeruginosa* PAO1, PA14, PAET1, PAET2 and PAET4 to A549, 16HBE14o- and CFBE41o- cells. The different plots show the log_10_ CFUs of each strain counted as adhered (adhered + invaded) and as only intracellularly invaded per monolayer of the different epithelial cells. Three independent experiments are plotted in each graph, and the error bars indicate the standard error of the mean from representative triplicate experiments. Statistical significance between the adhered and the invaded log_10_ of each strain CFU/monolayer point is indicated with an asterisk (*p < 0.05; ** p < 0.01; *** p < 0.001).

The interactions of *P. aeruginosa* strains with the different lung epithelial cells were examined. Two laboratory reference *P. aeruginosa* strains (PAO1 and PA14) along with three different clinical isolates from CF patients (PAET1, PAET2, and PAET4) were used in this study. After infection of A549, 16HBE14o- and CFBE41o- monolayers with the different *P. aeruginosa* strains for 3 h at a multiplicity of infection (MOI) = 100, the adhesion patterns of the PAO1, PA14, PAET1, PAET2, and PAET4 strains showed no significant changes (*p* > 0.05) when adhering to the same epithelial cell type (Figure 1 and Table S1). Major differences were observed when comparing the adhesion of the different *Pseudomonas* strains to A549 monolayers with their adhesion to 16HBE14o- or to CFBE41o- monolayers, as adhesion of some *P. aeruginosa* strains to A549 cells were detected significantly higher (Table S1). Specifically, and according to the statistical analysis done (Table S1), the reference PAO1 and PA14 strains exhibited higher adhesion to A549 monolayers than to 16HBE14o- and to CFBE41o- monolayers (*p* < 0.01). Similarly, the clinical isolates PAET2 and PAET4 showed greater adhesion to A549 monolayers than to CFBE41o- monolayers (*p* < 0.05), while PAET1 adhesion to A549 monolayers was also detected higher than to 16HBE14o- monolayers (*p* < 0.05; Figure 1 and Table S1).

Otherwise, significant differences in cell invasion were observed among the strains used depending on the type of lung epithelial cell that they were infecting. Remarkably, the clinical isolates showed an ~1.5–2 log_10_ higher capacity to invade 16HBE14o- and CF-related CFBE41o- cells than that of the *P. aeruginosa* reference PAO1 and PA14 strains (Figure 1), with invasion rates of ~20-50% and intracellular CFUs of ~1x10^4^-1x10^5^ CFU/monolayer. Conversely, the reference PAO1 and PA14 strains had invasion rate percentages of ~0.39–3.78% for 16HBE14o- and CFBE41o- cells (Table 1). In contrast, in A549 cells, the intracellular *Pseudomonas* CFUs were ~10^6^ CFU/monolayer among the different strains except for PA14 (Figure 1), and the highest invasion rate was calculated for the clinical isolate PAET1 (21.76%). PAO1 and the CF isolates PAET2 and PAET4 had similar invasion rates, ~4.0–6.96% (Table 1). As expected, the cytotoxic *P. aeruginosa* PA14 strain had the lowest invasion capacity, with invasion rates of 0.39–0.79% among the three epithelial monolayers.

**Table 1.** Percentage (mean±SD) values of the *P. aeruginosa* invasion rate per lung cell after 3 h of infection at a MOI = 100.

	A549	16HBE14o^−^	CFBE41o^−^
PAO1	5.50 (±3.15) %	3.78 (±1.05) %	3.24 (±1.01) %
PA14	0.77 (±0.18) %	0.39 (±0.08) %	0.79 (±0.18) %
PAET1	21.76 (±5.55) %	19.31 (±2.09) %	50.05 (±10.51) %
PAET2	6.96 (±1.91) %	37.81 (±3.98) %	18.07 (±3.06) %
PAET4	4.04 (±0.47) %	40.88 (±5.47) %	31.33 (±6.28) %

From these experiments, we could conclude that while similar adhesion properties were observed among the different *Pseudomonas* strains, the clinical CF isolates demonstrated a slightly increased invasion ability toward bronchial 16HBE14o- and CF- CFBE41o- cells than in the A549 cells. Otherwise, the reference PAO1 and PA14 strains reflected reduced capacity to invade both bronchial cells than invade A549 monolayers.

### *Higher persistence of intracellular CF-isolated* Pseudomonas *in 16HBE14o- and CFBE41o- cells*

**Figure 2.**
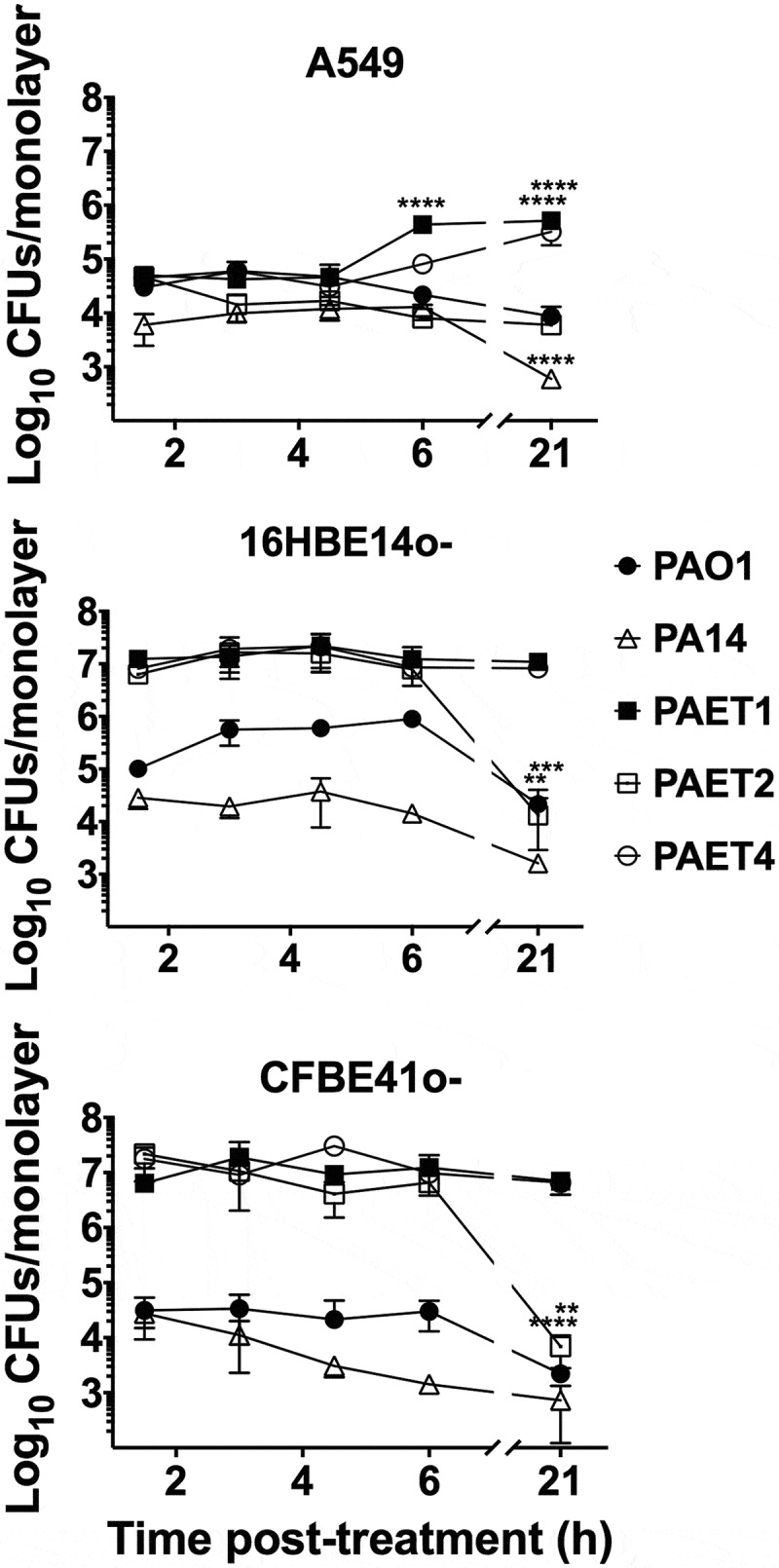
Differential pattern of intracellular persistence among the *Pseudomonas* strains depending on the lung epithelial cell type. The figure shows the log_10_ CFUs/monolayers of PAO1, PA14, PAET1, PAET2, and PAET4 after 1.5, 3, 4.5, 6 and 21 h incubation with gentamicin 200 µg/mL after 3 h of infection at a MOI = 1. The standard error of the mean from representative triplicate experiments is indicated with the error bars. Significant differences between the CFUs counted per monolayer between time points for each strain and cell line are indicated with asterisks (*p < 0.05; ** p < 0.01; *** p < 0.001; **** p < 0.0001).

Once cellular invasion was confirmed, we next analyzed the intracellular persistence among the different *Pseudomonas* strains within the three types of lung epithelial cells. This experiment was performed by initially infecting epithelial cells at a MOI = 1 for 3 h instead of a MOI = 100 to avoid rapid saturation of the cell monolayers. The intracellular *P. aeruginosa* CFUs were counted after 1.5 h, 3 h, 4.5 h, 6 h and 21 h of incubation with gentamicin (Gm) treatment (see Figure 2). The survival slope of each strain was also calculated to confirm the respective intracellular tendency inside the different alveolar and bronchial cells, as well as the percentage of intracellular CFU/monolayer at the 21 h time point compared to 1.5 h incubation with Gm (Table S2).

The results clearly showed the high persistence of the *P. aeruginosa* CF isolates PAET1, PAET2, and PAET4 inside the bronchial 16HBE14o- and CF-affected CFBE41o- cells, with intracellular CFUs maintained at ~1x10^7^ CFUs/monolayer over the course of the experiment (Figure 2). PAET1 strain reflected a 108.39% and 87.51% of the increase in CFU/monolayer after 24 h of intracellular infection of 16HBE14o- and CFBE41o-, respectively, while that increase was calculated of 37.07% and 101% in the PAET4 during infection of the same cells (Table S2). Only PAET2 showed a significantly decreased persistence at the 21 h time point, with CFUs of ~1x10^4^ per monolayer of 16HBE14o- and CFBE41o- bronchial cells (*p* < 0.001), corresponding to a 0.03% and 0.21% of survival with slopes calculated significantly non-zero (Table S2). In these two cell lines, the *P. aeruginosa* PAO1 and PA14 reference strains were able to persist intracellularly, although at lower CFUs per monolayer. Within 16HBE14o- cells, the CFU of PAO1 remained ~1x10^5^ over the initial 6 h with Gm treatment, but a significant decrease (*p < *0.01) was detected at the 21 h time point. A similar persistence pattern was observed for this reference strain inside cystic fibrosis CFBE41o- cells, although fewer CFUs were counted intracellularly: ~1x10^3^ -~1x50^4^ CFU/monolayer (Figure 2). The survival percentages of the PAO1 strain at 24 h time point were calculated of 7.01% and 21.31% inside of 16HBE14o- and CFBE41o- cells, respectively (Table S2). Otherwise, the CFUs of PA14 were ~1x10^4^ CFU/monolayer, with a gradual decrease to ~1x10^3^ CFU/monolayer at the final time point in both bronchial cell lines (Figure 2). In this reference strain, the survival percentage in CFU/monolayer after 24 h of infection (21 h of Gm treatment) was calculated of 2.63% and 5.69% inside 16HBE14o- and CFBE41o- monolayers (Table S2).

On the other hand, within A549 cells, the different *P. aeruginosa* strains showed a similar intracellular persistence during the initial 3 h of intracellular infection, and significant differences were detected from that time point on (Figure 2). The intracellular CFUs/monolayer were ~1x10^3-^ ~1x10^5^ in all strains over the course of the experiment except for PAET1, which showed a significant CFU increase at the 6 h time point (*p* < 0.001) that was maintained until the 21 h time point (Figure 2). For this clinical isolate, the survival percentage was calculated of 1030% after 24 h of intracellular persistence inside A549 cells with a positive slope determined significantly non-zero (Table S2). The PAET4 strains also reflected a significant increase of CFU/monolayer at this final time point calculated of the 719.37% (Figure 2 and Table S2). In these alveolar cells, the clinical isolate PAET2 behaved similar than the reference PAO1 and PA14 strains, with survivals calculated for the three *P. aeruginosa* strains between the 9.98% and 29.29% (Table S2).

**Figure 3.**
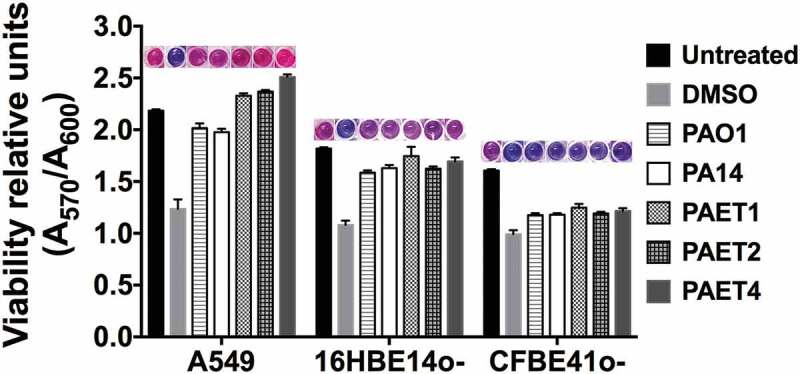
Intracellular persistence of the *P. aeruginosa* reference and clinical strains does not affect lung cell viability. A549, 16HBE14o- and CFBE41o- toxicity after 24 h of intracellular persistence of PAO1, PA14, PAET1, PAET2, and PAET4. The plot shows the normalized values of the absorbance measured at A_570_ to the absorbance of the reference A_600_, as recommended by the PrestoBlue^TM^ manufacturer. Higher absorbance values indicate greater metabolic activity and therefore viability. Data from three independent experiments are shown, and the error bars indicate the standard error of the mean of representative triplicate experiments. Untreated monolayers were used as controls for viability, and DMSO-treated cells were used as controls for toxicity. A representative picture of the Prestoblue^TM^-treated cells is included in the figure in which the gradient from pink (viable cells) to dark blue (toxic cells) is related to the level of cellular toxicity.

In summary, these results demonstrate that while the reference strains showed similar intracellular persistence patterns among the three lung epithelial cell lines, the clinical isolates of *P. aeruginosa* had an increased tendency to invade and persist inside the different bronchial cells. In some cases, intracellular replication of some *Pseudomonas* strains could also be detected in addition to just persistence, as it was the case of PAET1 and PAET4. Nevertheless, when cytotoxicity was assessed to ensure that the CFU counted inside the epithelial cells after 21 h of Gm treatment come from viable cells, no significant changes (*p > *0.05) were detected between the 21 h Gm-treated infected monolayers and untreated cells (Figure 3). Although some monolayers contained apoptotic cells with clear nuclear condensation, no major destruction was observed, and the three different lung cells retained their confluency (Figure S2). Only treatment with DMSO promoted a loss of viability and cellular destruction (Figure 3 and Figure S2).

### *Differential oxygen consumption among the lung cells depends on the type of infectious* P. aeruginosa *strain*

**Figure 4.**
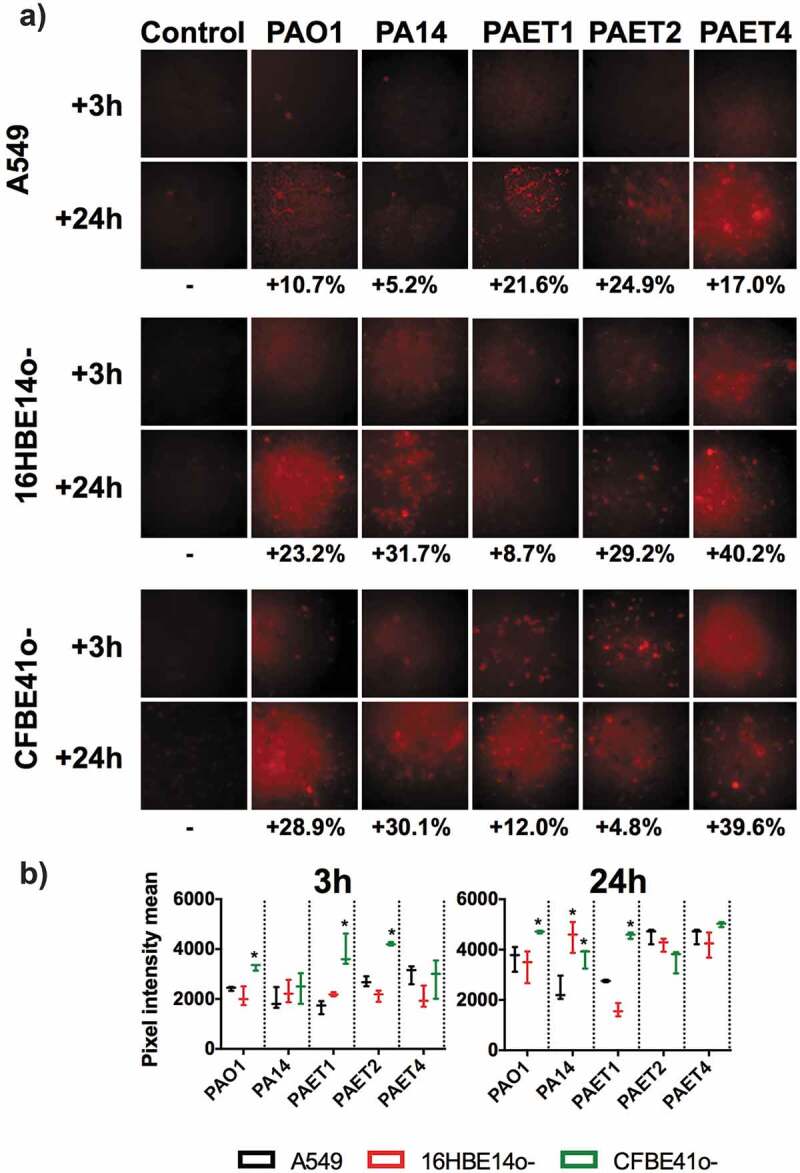
Oxygen consumption of A549, 16HBE14o- and CFBE41o- *P. aeruginosa-*infected monolayers. A) Fluorescence microscope images showing the red fluorescence emission of the hypoxia probe dye after 3 and 24 h of initial infection of the PAO1, PA14, PAET1, PAET2 and PAET4 strains. Uninfected A549, 16HBE14o- and CFBE41o- were also included as controls. The red-intensity signal relates to cellular hypoxia. The percentage of red intensity increases from 3 h to 24 h of intracellular persistence per infection and lung epithelial cell type and is included in the figure. The percentages were calculated with ImageJ software using the pixel intensity averages from ten different images of each infectious condition and time-point. B) Graphs showing the pixel intensity averages and the respective standard error of the mean of each *P. aeruginosa* intracellular infection of A549, 16HBE14o- and CFBE41o- cells incubated for 3 and 24 h. Significance differences among lung cell infections with the same *P. aeruginosa* strains are denoted with asterisks (*) *p > *0.05.

Oxygen is a critical element for aerobic metabolism and biofilm formation [19]. In contrast to eukaryotic organisms, some prokaryotes can modulate their metabolism and live under anaerobic conditions [20]. In tissues, hypoxia is a symbol of damage, inflammation, and infection [10]. Therefore, the oxygen consumption promoted by infection of the different lung epithelial monolayers with the *Pseudomonas* strains was examined. For the examination of oxygen consumption, the levels of intracellular oxygen after 3 and 24 h of monolayer infection were analyzed using a hypoxia probe (Figure 4). Uninfected A549, 16HBE14o- and CFBE41o- monolayers were included as controls.

Red intensity quantification revealed that all of the uninfected lung epithelial monolayers did not increase the respective hypoxic state between 3 and 24 h (*p* > 0.05, Figure 4). The red intensity quantified in the different A549, 16HBE14o- and CFBE41o- infected monolayers was normalized with that detected in the respective uninfected-control cell. The difference in red intensity between 3 and 24 h of each cell infection was subsequently calculated as relative to oxygen consumption. An increase in cellular anaerobiosis was detected in all infected cells, independently of the *P. aeruginosa* strain, after 3 and 24 h (Figure 4a,b). This increase was calculated to be between 5.2% and 39.6% (Figure 4a). Generally, the increase in red intensity promoted by a 3 h infection of the CF-derived CFBE41o- monolayers was detected to be higher than that observed in the other lung epithelial cells regardless of the *Pseudomonas* infection type (Figure 4a,b). Significantly, a higher increase in anaerobiosis (red intensity) was detected after 3 h in CFBE41o- monolayers infected with PAO1, PAET1, and PAET2 compared to that caused by infection of the other cells (*p* < 0.05). This increase was calculated to be between ~1.5- and ~2.30-fold compared to that caused by the other infection types (Figure 4b). Among the intracellular infections incubated for 24 h, the anaerobic state promoted by PAO1 and PAET1 in CFBE41o- cells was significantly increased (*p* < 0.05) compared to that caused by PAO1 and PAET1 in A549 or 16HBE14o- cells. These increases were calculated to be ~1.37- and 1.53-fold higher for PAO1 infection of CFBE41o- compared to PAO1 infection of A549 and 16HBE14o-, respectively, and ~1.73- and 2.74-fold higher for the same comparisons but with PAET1 (Figure 4b). Furthermore, PA14 infection of both 16HBE14o- and CFBE41o- cells also led to higher oxygen consumption than that after infection of A549 cells (Figure 4a,b).

This experiment confirmed differential oxygen consumption intracellularly between the different A549, 16HBE14o-, and CFBE41o- infections with the reference and clinical isolates of *P. aeruginosa.*

### *The intracellular environment modulates* Pseudomonas *RNR expression over time*

The *P. aeruginosa* reference strains and the clinical isolates promoted different oxygen consumption and cellular hypoxia levels among the three lung epithelial cells. Furthermore, the different *P. aeruginosa* strains were able to persist within the A549, 16HBE14o-, and CFBE41o- cells, thus indicating their capacity for DNA replication in this oxygen-changing environment. As ribonucleotide reductase (RNR) plays a key role in the synthesis of the dNTPs required for DNA replication, we next studied its expression after 3 and 24 h of monolayer infection.

**Figure 5.**
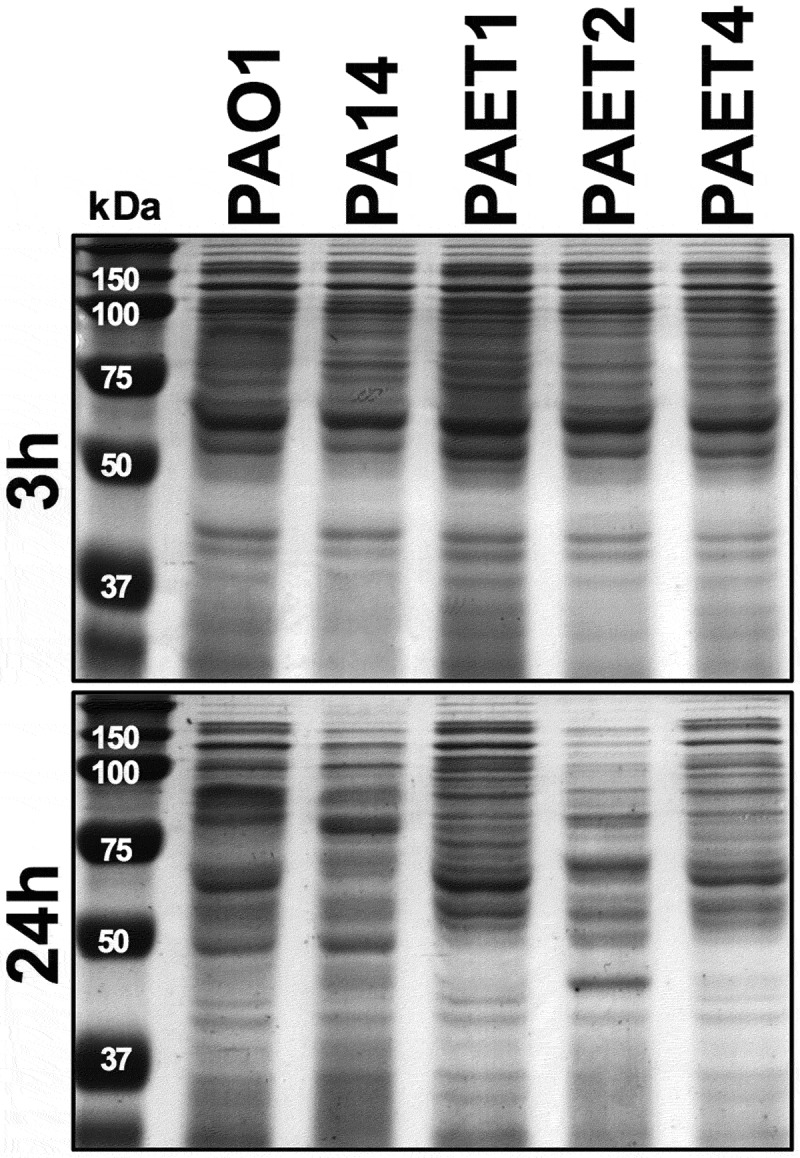
Shifting protein expression pattern among the reference and CF isolates of *P. aeruginosa*. The figure shows the picture of two SDS-PAGE gels with the protein extracts of the PAO1 and PA14 reference strains together with the PAET1, PAET2 and PAET4 CF-clinical isolates after 3 and 24 h of planktonic growth. While all *P. aeruginosa* strains shared similar protein pattern after 3 h of growth, it completely changed after 24 h, with a completely different protein expression profile between strains independently their background.

Since the *P. aeruginosa* strains used in this work came from different backgrounds, the protein expression pattern of each strain was analyzed at the chosen time points to evaluate RNR expression. Thus, the protein expression profiles of PAO1, PA14, PAET1, PAET2, and PAET4 were examined after 3 and 24 h of growth (Figure 5). Interestingly, while all the strains showed a similar protein expression pattern after 3 h of growth (exponential phase), the protein expression patterns completely changed at the 24 h time point. After 24 h of bacterial growth, the picture was different, and only the clinical isolates PAET1 and PAET4 had similar protein band profiles to those detected at the 3 h time point. PAO1, PA14, and PAET2 showed completely different protein expression patterns between the two time points (Figure 5).

**Figure 6.**
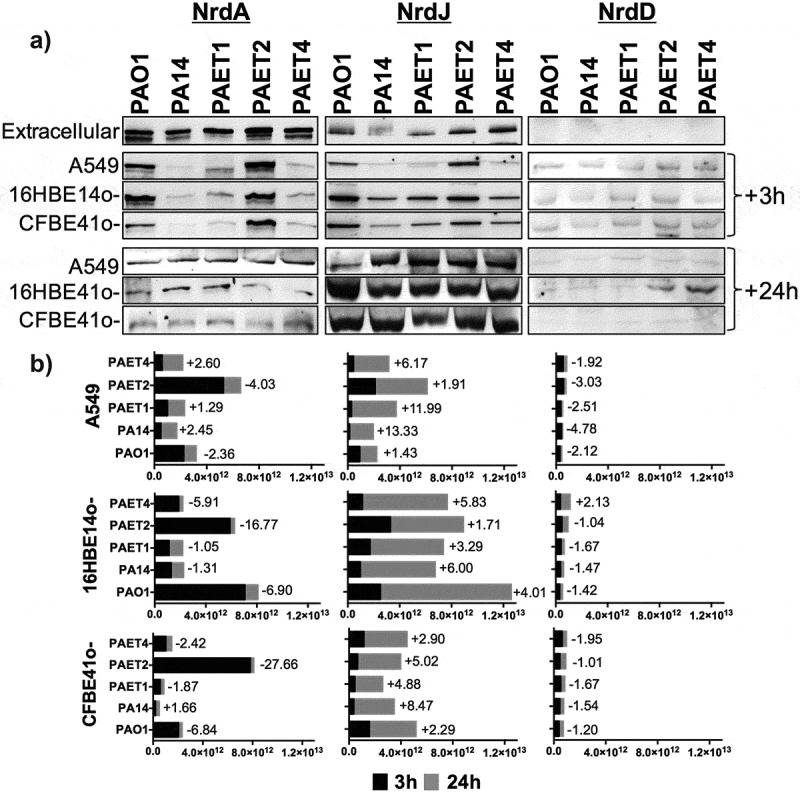
Different lung intracellular backgrounds promote differential RNR expression among *P. aeruginosa* strains. A) Intracellular NrdA, NrdJ and NrdD protein expression in *P. aeruginosa* PAO1-, PA14-, PAET1-, PAET2- and PAET4-infected A549, 16HBE14o- and CFBE41o- cells after 3 and 24 h are shown in the figure. The levels of extracellular *P. aeruginosa* NrdA, NrdJ and NrdD from the same strains grown in the extracellular phase (media) while infecting are also included. Specific NrdA, NrdD and NrdJ protein bands were selected according their molecular weight as given by antibodies binding to purified NrdA, NrdD and NrdJ proteins from our laboratory stock (Figure S1A). Unspecific bands of each independent Nrd protein immunoblot were used as loading controls, and the detection of purified NrdA (~107.1 kDa), NrdJ (~82.7 kDa) and NrdD (~76.1 kDa) proteins with polyclonal antibodies was used as the Nrd protein band detection control (Figure S1B). B) The different plots show the average of the number of pixels calculated in the volume of NrdA, NrdJ and NrdD protein bands using ImageQuant^TM^ LAS4000 software. A higher pixel intensity in the protein band volume indicates higher expression of the target protein under a specific infection condition. Each plot compares the Nrd protein bands detected at 3 and at 24 h of intracellular persistence inside of A549, 16HBE14o- and CFBE41o- monolayers and includes the fold induction of each protein at the 24 h time-point. Protein induction was calculated using the average of the pixels determined from each protein band at both time-points, previously normalized by the average of the pixels determined in the respective unspecific band shown in (Figure S1B).

Extracellular detection of the ribonucleotide reductase protein (Nrd) showed a clear common pattern among the different *P. aeruginosa* strains, in which NrdA (class Ia RNR) and NrdJa (class II RNR; referred to NrdJ from this point onwards) dominated extracellular expression. However, NrdJ was detected at lower levels than NrdA protein. As expected, NrdD protein (class III RNR) was not detected under aerobic conditions in any of the *P. aeruginosa* strains (Figure 6a). Western blot analysis revealed that intracellular NrdA and NrdJ expression changed among the *P. aeruginosa* strains, although a common pattern was found among the different infected lung epithelial monolayers. Furthermore, for these two RNR proteins, the expression profile changed between 3 and 24 h, with a clear induction of NrdJ, while NrdA tended to decrease during intracellular persistence within lung epithelial cells (Figure 6a,b). Clearly, the PAO1 and PAET2 strains showed higher NrdA expression after 3 h of intracellular infection than that of PA14, PAET1, and PAET4, independently of the type of infected lung epithelial cell. These two groups of *Pseudomonas* had a similar intracellular pattern of NrdA expression at the 3 h time point. Protein band quantification and average pixel calculations determined that NrdA expression in the PAO1 was ~1.98–11.01-fold higher than that detected in PA14, PAET1, and PAET4 in infected cells of the three epithelial lung cell lines. On the other hand, NrdA induction in the PAET2 strain, compared to the other three *P. aeruginosa* strains after 3 h of infection of A549, 16HBE14o-, and CFBE41o- was calculated of ~3.14–42.36-fold (Figure 6a,b and Supplementary Table S3). However, after 24 h of intracellular persistence, the different *P. aeruginosa* strains had similar NrdA expression within alveolar and bronchial cells, revealing similar protein expression patterns (Figure 6a). In that sense, the majority of *P. aeruginosa* strains suffered a reduction of ~1.05- to ~27.66-fold in NrdA expression during 24 h of intracellular persistence (Figure 6b). Significantly, protein band quantification determined that NrdJ expression by the different *P. aeruginosa* strains increased from ~1.43- to 13.33-fold after 24 h of intracellular persistence. In the *P. aeruginosa* PAO1 reference strains, the expression of NrdJ increased by ~1.43-, 4.01-, and 2.29-fold after 24 h of intracellular infection of A549, 16HBE14o-, and CFBE41o- cells, respectively. Otherwise, induction of NrdJ expression between the two time points was significant in the other reference strain, *P. aeruginosa* PA14, with protein inductions of ~13.33-, ~6.00- and ~8.47-fold for the same cell infections. Among the *P. aeruginosa* clinical isolates, the PAET1 and PAET4 strains showed higher intracellular induction of NrdJ expression than that in PAET2, although increased levels of the protein were also detected after 24 h in PAET2 intracellular infection (Figure 6a,b). Specifically, in the PAET1 strain, NrdJ expression increased by ~11.99-, ~3.29- and ~4.88-fold after 24 h of intracellular persistence in A549, 16HBE14o-, and CFBE41o- cells, respectively. PAET2 revealed a class II RNR protein induction of ~1.91-, ~1.71- and ~5.02-fold for the same cell infections and intracellular persistence time, while in the clinical PAET4, NrdJ induction was calculated to be ~6.17-, ~5.83- and ~2.90-fold higher after 24 h of intracellular persistence within the different lung epithelial monolayers.

On the other hand, no major differences were detected in the expression of the NrdD protein between the different *P. aeruginosa* strains. The expression of this RNR protein was higher after 3 h of infection than after 24 h, and only PAET4 demonstrated a ~ 2.13-fold induction of NrdD expression after 24 h of intracellular persistence in 16HBE14o- cells (Figure 6a,b). Interestingly, the induction in NrdD expression in PAET4 coincided with the infection condition that caused the highest increase in cellular hypoxia (~40.2%) after 24 h of intracellular persistence, as shown in Figure 4.

These results indicate an important role of class II RNR in providing the dNTPs necessary for DNA homeostasis during intracellular *P. aeruginosa* infection of lung epithelial cells, for which different times may be required, depending on the *P. aeruginosa* strain, to shift from the extracellularly expressed class Ia RNR to the class II RNR.

### *Intracellular modulation of the* nrd *genes between the reference and clinical P. aeruginosa strains leads to their essential role during the intracellular survival of the strain*

**Figure 7.**
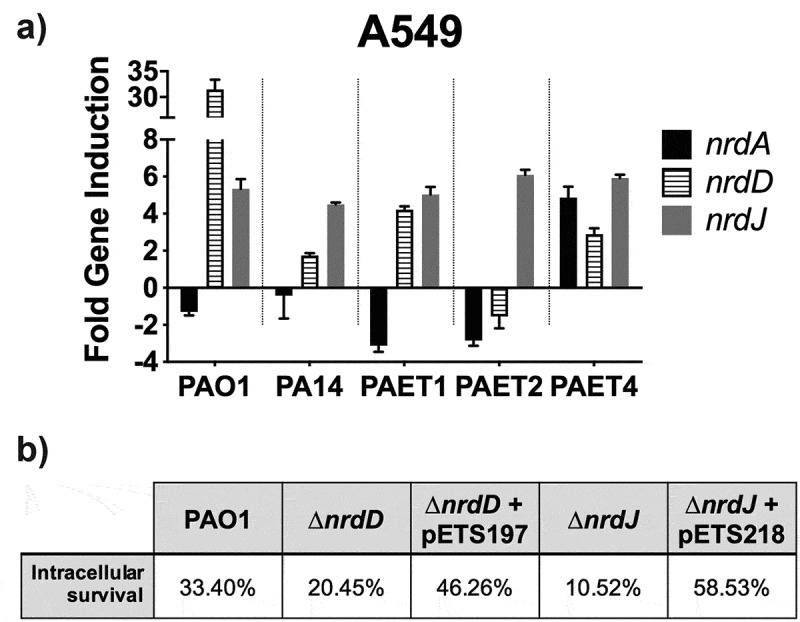
Intracellular expression of the *P. aeruginosa*’s *nrd* genes and their importance during the persistence of the bacterium inside the A549 cells. A) Fold-change induction of *nrdA, nrdJ and nrdD* gene expression in PAO1-, PA14-, PAET1-, PAET2- and PAET4-infected A549 after 24 h of intracellular infection compared to 3 h. The *nrd* gene induction was calculated relative to the endogenous control *gapA* and using the 2^−ΔΔCT^ method. B) Percentages of intracellular survival for the PAO1 WT, PAO1 Δ*nrdJ*, PAO1 Δ*nrdD* and the respective PAO1 Δ*nrdJ+*pETS218 and PAO1 Δ*nrdD*+pETS197 complemented strains after 21 h of Gm treatment. Cb 300 μg/mL was included during the experiment in the conditions with the complemented strains to ensure plasmid maintenance.

To confirm the results of RNR’s intracellular protein expression in the different reference strains and clinical isolates of *P. aeruginosa*, qRT-PCR of *nrdA, nrdD* and *nrdJ* genes were performed after 3 and 24 h of persistence within the A549 cells. Since, the tendency of RNR’s expression in the strains was similar between the different lung epithelial cells (Figure 6), this experiment was performed only in the A549 cells. Results indicated a similar expression pattern between the reference PAO1 and PA14 strains together with the PAET1 clinical isolate (Figure 7a). In these three strains, the *nrdD* and *nrdJ* genes reflected a clear intracellular induction, whereas *nrdA* expression was repressed. Inductions for the *nrdD* gene after 24 h of intracellular infection were calculated ~32 fold in the PAO1, ~1.8 fold in the PA14 reference strain and ~4.2 in the clinical PAET1 isolate. Otherwise, *nrdJ* expression showed ~5–7 fold induction in these three *P. aeruginosa* strains. On the other side, the PAET2 and PAET4 clinical isolates reflected a different and unique, *nrd* gene expression pattern. While the clinical PAET2 strain only showed intracellular induction for the *nrdJ* gene, which was calculated ~6 fold increased at 24 h compared to 3 h infection, the PAET4 isolate induced the three *nrdA, nrdD* and *nrdJ* genes during the 24 h of persistence. 24 h expression of *nrdA* gene was calculated >4 fold compared to 3 h infection, while *nrdD* and *nrdJ* inductions were calculated ~3 and ~6 fold, respectively, in the PAET4 during the intracellular infection (Figure 7a).

To finally demonstrate the importance of classes II and III RNR during the intracellular survival of *P. aeruginosa*, we evaluated the ability to persist off the Δ*nrdJ* and Δ*nrdD* isogenic mutants inside the A549 cells, compared to their PAO1 WT strains in the same conditions and time points as shown in Figure 2. The complemented strains for the PAO1 Δ*nrdJ* and PAO1 Δ*nrdD* were constructed and included in the experiment. After 24 h of intracellular persistence, both PAO1 Δ*nrdJ* and PAO1 Δ*nrdD* mutants reflected less intracellular survival than that observed by the PAO1 WT strain, with the percentage of intracellular survival after 24 h calculated ~20.45% for the *nrdD* mutant and ~10.52% for the Δ*nrdJ* strain (Figure 7b). When the respective mutations were complemented, intracellular survival increased to ~46.26% and ~58.53% for PAO1 Δ*nrdD* and PAO1 Δ*nrdJ* respectively. On the other hand, the PAO1 strains showed similar persistence than that shown in Figure 2, with final intracellular survival calculated ~33.40% (Figure 7b).

Overall, these results confirm the critical role of class II RNR during the intracellular survival of *P. aeruginosa* strains.

## Discussion

In this study, we focused on the analysis and understanding of the chronicity of lung infections promoted by the intracellular persistence of *P. aeruginosa*. Using two *P. aeruginosa* reference strains and three different clinical isolates, we confirmed differential infectious behaviors depending on the *P. aeruginosa* background, indicating a directed specialization of the clinical isolates to infect a particular type of lung epithelial cell. Evaluations of the intracellular oxygen levels in basal uninfected alveolar (A549) and bronchial (16HBE14o- and CFBE41o-) cells as well their specific and independent changes during infection with the different *Pseudomonas* strains were also analyzed. Significantly, our investigations revealed that expression of class II RNR is highly induced during *P. aeruginosa* intracellular infection and is thus the major dNTP supplier during intracellular persistence of this pathogen.

Intracellular invasion is an advantageous mechanism used by *P. aeruginosa*, among many other pathogens, to evade the host immune system and antimicrobial therapy, which consequently promotes infection persistence and recurrence [21]. *P. aeruginosa* has been shown to invade nonphagocytic cells, such as endothelial or epithelial cells. Successful invasion is mediated by cytoskeletal rearrangements of eukaryotic host cells by the modulation of host actin and microtubule dynamics [22,23]. At that level, *P. aeruginosa* expresses different virulence factors to inject different toxins as well as to facilitate cellular uptake [7]. Adhesion to and subsequent invasion of the different A549, 16HBE14o-, and CFBE41o- epithelial cells were detected among the different *P. aeruginosa* strains regardless of the background of the strains. After invasion, the reference *P. aeruginosa* PAO1 and PA14 strains in addition to the different PAET1, PAET2 and PAET4 CF isolates showed the capacity to persist intracellularly, with detection of bacterial growth occurring with the PAET1 and PAET4 *P. aeruginosa* clinical strains within the A549 cells (Figures 1 and 2). However, the invasion rates and the levels of intracellular persistence and replication were found to be cell- and strain-dependent. The invasion capacity of the *P. aeruginosa* strains within host cells *in vitro* notwithstanding the *P. aeruginosa* phenotype have been broadly observed [7,17,21,23]. It should be noted that there is heterogeneous genotypic clonality among *P. aeruginosa* infections as well as different protein expression profiles (including important virulence factors) that depend on the *P. aeruginosa* background [4,24]. In this sense, large portions of DNA in the genomes of these *P. aeruginosa* clinical strains are missing in the reference and well-studied PAO1 strain [1,2,9]. Our study revealed a shifting protein expression pattern across the different reference and clinical *P. aeruginosa* strains, which was found to change over time. Thus, while a similar protein profile was detected after 3 h of growth, it completely changed by 24 h, with no correlation with the original bacterial background (Figure 5). Significantly, adapted CF-*Pseudomonas* undergo pathoadaptative mutations, genomic rearrangements and deletions that are evolutionarily selected to be beneficial to survive in the CF lung environment. These adaptive mutations and genomic rearrangements evolve differently depending on the patient [4,24]. In agreement with these observations, our results showed the clear efficiency of the CF isolates to persist and replicate intracellularly in bronchial 16HBE14o- cells and their CF-derived CFBE41o- cells.

Since a similar behavior among the different *P. aeruginosa* strains was detected within adenocarcinomic A549 cells, it is possible that the CF isolates PAET1, PAET2, and PAET4 encode similar mutations and have other similarities in their genome that favor their persistence within the bronchial epithelium that could be lacking in the PAO1 or PA14 genomes. Therefore, the possible mutations or genomic rearrangements occurring in previous infections may have allowed the clinical isolates to develop the most successful invasion capacity. Furthermore, the CFTR protein has been postulated to be important for *P. aeruginosa* internalization inside lung epithelial cells [25], which supports the idea that the clinical CF isolates PAET1, PAET2, and PAET4 may have undergone directed adaptation to allow them to colonize CFBE41o- cells, as we have detected in our work. Inside the cell, *P. aeruginosa* can persist and replicate intracellularly, as detected in our study as well as in other cellular models [7,17,25]. Bacterial persistence within host cells can constitute a latent reservoir that is helped by the poor intracellular penetration of some antibiotics and can lead to the selection of antibacterial resistant mutants and contribute to disease harshness and chronicity [7,21].

Studies have indicated that *P. aeruginosa* grows in cystic fibrosis lungs via aerobic respiration, yet the mucus plugs present in the CF airways are majorly anoxic microenvironments [26]. Furthermore, cellular hypoxia is usually associated with infection, injured tissue, or inflammation and plays an important role in the host immune response [10]. Specifically, for *P. aeruginosa* infections, hypoxia has been shown to modulate infection in epithelial cells [27]. In this work, different basal levels of cellular oxygen were detected in pulmonary epithelial cells, with a higher state of hypoxia detected in CFBE41o- cells than in 16HBE14o- or A549 cells. Additionally, the CF isolates mediated faster oxygen depletion when infecting CFBE41o- monolayers (Figure 4). ΔF508 CFTR cells have altered lipid homeostasis, which can induce cellular death and apoptosis [28]. Furthermore, CFBE41o- cells express lower levels of heme oxygenase-1 (HO-1), a stress-inducible protein with anti-inflammatory and antioxidant properties that are induced by signals mediated through LPS binding to Toll-like receptor 4 (TLR4). Nevertheless, CFBE14o- cells have an impaired expression of TLR4 at the cellular surface. The lack of HO-1 is known to promote iron accumulation inside CFBE41o- cells, which leads to cellular hypoxia with abnormal activity of hypoxia-inducible factor 1α (HIF-1α) [29]. The absence of HO-1 has also been linked to continuous inflammation and cellular damage state in mice [30], which may explain the higher basal toxicity detected in CFBE41o- monolayers (Figure 3). It is possible that this increased toxicity may influence the intracellular persistence of the bacterium. However, in addition to the basal oxygenic state of the cells, a common trait among the different infectious conditions was the differential oxygen consumption during the intracellular persistence of the different *P. aeruginosa* strains within the A549, 16HBE14o-, and CFBE41o- monolayers, which is in agreement with other studies, indicating that pathogen colonization of the lung epithelium exacerbates tissue hypoxia [31] by increasing their own metabolism [18].

Intracellular lifestyles, as all types of lifestyle, require a continuous dNTP supply to survive, and the RNR is the unique enzyme capable of providing *de novo* dNTPs [6]. In this paper, we found that the intracellular lifestyle drives *P. aeruginosa* to express class II RNR (NrdJ) as the dNTP supplier. High NrdJ induction was detected in all strains regardless of the type of epithelial cell they were infecting. However, at earlier infection times, higher NrdJ protein expression was detected during *Pseudomonas* intracellular infection of bronchial cells than A549 cells (Figure 6). As an indispensable requisite, class II RNR depends on vitamin B_12_ or 5ʹ-deoxyadenosylcobalamin (AdoCbl) to act as an external cofactor to perform its enzymatic activity. Vitamin B_12_ was included in the formulation of the cell culture media used in this study. People with CF supplement their diet with vitamin B_12_ due to their malabsorption of fat- and water-soluble vitamins as a consequence of pancreatic enzymes and bile salt deficiencies, which increase the levels of this vitamin in serum [32]. We have recently demonstrated higher levels of intracellular vitamin B_12_ in the CF-isolate PAET1 compared to those in the reference PAO1 strain [33]. Previous studies detected the induction of class III RNR and class II RNR during *P. aeruginosa* infection of *Drosophila melanogaster* [5] and of *Danio rerio* [14], in the present study, induction of *nrdD* expression was detected at the gene expression level but not at the protein level. NrdD was only moderately overexpressed in the CF-isolate PAET4 after 24 h of intracellular persistence of 16HBE14o- cells, which is the infectious condition that promoted higher induction of hypoxia. Class III RNR expression in *P. aeruginosa* has been shown to be increased under anaerobic conditions with the subsequent repression of class I and II RNRs [14]. Although hypoxia was confirmed to occur in the different infections, possible trace oxygen present in different intracellular compartments may prevent the activity of the anaerobic class III RNR. The oxidative stress present in the cellular compartments promoted by infections, as has been observed in CF-affected lungs, may induce the expression of class II RNR [34]. Hence, we hypothesize that the oxygen independence of class II RNR in addition to the stress present in the infectious environment favor the expression of this RNR class and the subsequent adaptation of *P. aeruginosa* to the intracellular lifestyle, which allows the formation of a bacterial reservoir and contributes to infection recurrence and chronicity.

Our study demonstrates the different pathophysiologies generated in different *in vitro* models of lung epithelium depending on the *P. aeruginosa* strain used. Therefore, the choice of bacterial strain is critical to properly study the type of infectious process with relevant translational outcomes. Furthermore, the heterogeneous expression of RNRs in the prokaryotic world opens a window in the treatment of infections. The exclusivity of class Ia RNR expression by humans allows the other RNR classes to be treated as antimicrobial targets. Different RNR classes have been postulated to be antimicrobial targets to combat infections. In this sense, science is moving toward the development of antimicrobial strategies against this enzyme [6,35]. In this work, we related class II RNR expression to the intracellular persistence of *P. aeruginosa* in different types of lung epithelial cells, which may contribute to the development of RNR-targeted strategies against the chronicity of this infection.

## Supplementary Material

Supplemental Material
