# Supplementary material for: Differential adaptability between reference strains and clinical isolates of Pseudomonas aeruginosa into the lung epithelium intracellular lifestyle
Source: Virulence. 2020 Jul 22;11(1):862–76. doi: 10.1080/21505594.2020.1787034 (PMC7549915; doi:10.1080/21505594.2020.1787034)
Supplement: Supplemental Material [file KVIR_A_1787034_SM3045.docx]

**Differential adaptability between reference strains and clinical isolates of *Pseudomonas aeruginosa* into the lung epithelium intracellular lifestyle**

Maria del Mar Cendra and Eduard Torrents

**Supplemental material**

**Figure S1. Controls used for the intracellular detection of the Nrd proteins. A)** *P. aeruginosa* Nrd proteins band detection using purified NrdA, NrdJa and NrdD proteins reacted with the antibodies used in this study. B) Unspecific bands of each immunoblot used as endogenous loading control of the western blot.


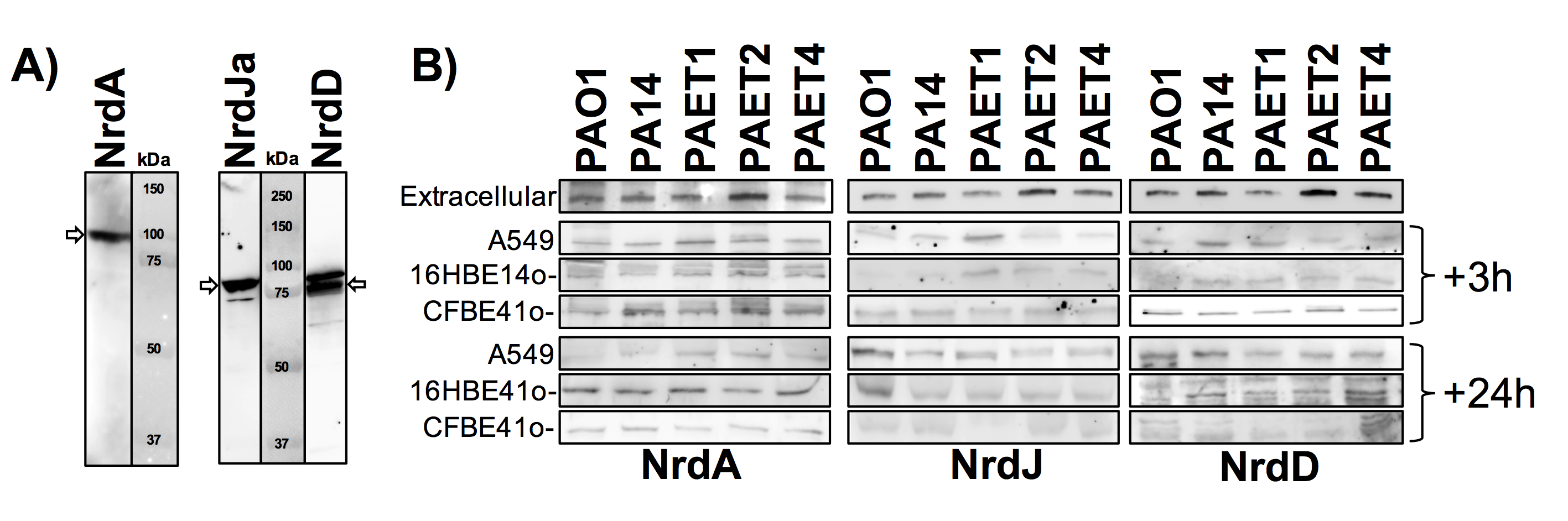


**Figure S2. Micrographs of the A549, 16HBE14o- and CFBE41o- after 24 h of intracellular persistence of the different reference strains and clinical isolates of *P. aeruginosa*.** Non-infected and DMSO-treated cells were used as negative and positive controls.


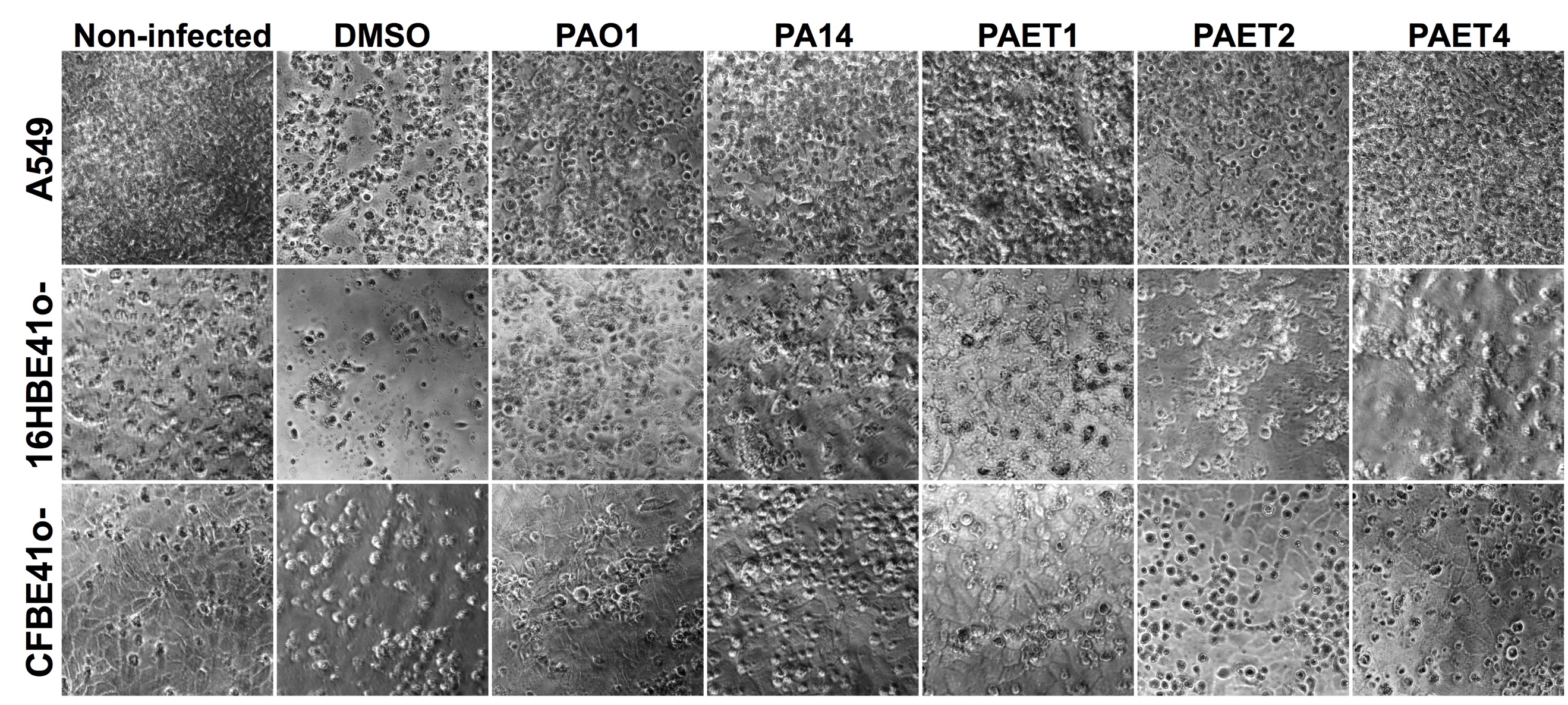


**Table S1.** Statistical significance of *P. aeruginosa* association with the different lung epithelial cells using a one way anova with tukey’s multiple comparison. Statistical significance is denoted with asterisks: **p*<0.05*,* ** *p*<0.01, *** *p*<0.001, NS: not significantly different.

|  | | | |  | **A549** | | | | |
| --- | --- | --- | --- | --- | --- | --- | --- | --- | --- |
|  | | | |  | PAO1 | PA14 | PAET1 | PAET2 | PAET4 |
| **A549** | | | | PAO1 |  | NS | NS | NS | NS |
|  |  |  |  | PA14 |  |  | NS | NS | NS |
|  |  |  |  | PAET1 |  |  |  | NS | NS |
|  |  |  |  | PAET2 |  |  |  |  | NS |
|  |  |  |  | PAET4 |  |  |  |  |  |
|  | |  | | | **16HBE14o-** | | | | |
|  | |  | | | PAO1 | PA14 | PAET1 | PAET2 | PAET4 |
| **16HBE14o-** | | PAO1 | | |  | NS | NS | NS | NS |
|  |  | PA14 | | |  |  | NS | NS | NS |
|  |  | PAET1 | | |  |  |  | NS | NS |
|  |  | PAET2 | | |  |  |  |  | NS |
|  |  | PAET4 | | |  |  |  |  |  |
|  | | |  | | **CFBE41o-** | | | | |
|  | | |  | | PAO1 | PA14 | PAET1 | PAET2 | PAET4 |
| **CFBE41o-** | | | PAO1 | |  | NS | NS | NS | NS |
|  |  |  | PA14 | |  |  | NS | NS | NS |
|  |  |  | PAET1 | |  |  |  | NS | NS |
|  |  |  | PAET2 | |  |  |  |  | NS |
|  |  |  | PAET4 | |  |  |  |  |  |
|  | | | |  | **16HBE14o-** | | | | |
|  | | | |  | PAO1 | PA14 | PAET1 | PAET2 | PAET4 |
| **A549** | | | | PAO1 | ** | ** | * | NS | NS |
|  |  |  |  | PA14 | ** | ** | NS | NS | NS |
|  |  |  |  | PAET1 | NS | NS | NS | NS | NS |
|  |  |  |  | PAET2 | NS | NS | NS | NS | NS |
|  |  |  |  | PAET4 | NS | NS | NS | NS | NS |
|  | | |  | | **16HBE14o-** | | | | |
|  | | |  | | PAO1 | PA14 | PAET1 | PAET2 | PAET4 |
| **CFBE41o-** | | | PAO1 | | NS | NS | NS | NS | NS |
|  |  |  | PA14 | | NS | NS | NS | NS | NS |
|  |  |  | PAET1 | | NS | NS | NS | NS | NS |
|  |  |  | PAET2 | | NS | NS | NS | NS | NS |
|  |  |  | PAET4 | | NS | NS | NS | NS | NS |
|  |  | | | | **CFBE41o-** | | | | |
|  |  | | | | PAO1 | PA14 | PAET1 | PAET2 | PAET4 |
| **A549** | PAO1 | | | | *** | *** | NS | * | * |
|  | PA14 | | | | ** | ** | NS | NS | NS |
|  | PAET1 | | | | NS | NS | NS | NS | NS |
|  | PAET2 | | | | NS | NS | NS | NS | NS |
|  | PAET4 | | | | NS | NS | NS | NS | NS |

**Table S2.** *P. aeruginosa* PAO1, PA14, PAET1, PAET2 and PAET4 intracellular survival slopes determined according the intracellular CFU/monolayers overtime shown in Figure 2. The table the P value to determine if the slope if significantly non-zero. The calculations were done using the Graphpad Prism software. Percentage of each *P. aeruginosa* strains survival within the A549, 16HBE14o- and CFBE41o- cells after 24 h of intracellular survival is also shown.

|  | **Strains** | **Slope** | **P value** | **Survival % after 24 h** |
| --- | --- | --- | --- | --- |
| **A549** | PAO1 | -1702.0 | 0.022 | 29.29% |
|  | PA14 | -823.3 | 0.322 | 9.98% |
|  | PAET1 | 24163.0 | 0.044 | 1030% |
|  | PAET2 | -431.0 | 0.402 | 12.93% |
|  | PAET4 | 17048.0 | 0.011 | 719.37% |
| **16HBE14o-** | PAO1 | -44870.0 | 0.028 | 7.01% |
|  | PA14 | -1655.0 | 0.159 | 2.63% |
|  | PAET1 | -443964.0 | 0.458 | 108.39% |
|  | PAET2 | -773556.0 | 0.016 | 0.03% |
|  | PAET4 | -490180.0 | 0.406 | 37.07% |
| **CFBE41o-** | PAO1 | -1371.0 | 0.221 | 21.31% |
|  | PA14 | -908.0 | 0.082 | 5.69% |
|  | PAET1 | -224459.0 | 0.449 | 87.51% |
|  | PAET2 | -324925.0 | 0.043 | 0.21% |
|  | PAET4 | -943649.0 | 0.150 | 101.% |

**Table S3.** NrdA protein band pixel average increase in PAO1 and PAET2 relative to PA14, PAET1 and PAET4 after 3 h intracellular infection of A549, 16HBE14o- and CFBE41o- cells.

|  |  | **PAO1 band-fold increase** | **PAET2 band-fold increase** |
| --- | --- | --- | --- |
| **A549** | PA14 | 4.53 | 10.64 |
|  | PAET1 | 2.24 | 5.25 |
|  | PAET4 | 3.74 | 8.80 |
| **16HBE14o-** | PA14 | 5.51 | 4.60 |
|  | PAET1 | 6.32 | 5.28 |
|  | PAET4 | 3.77 | 3.14 |
| **CFBE41o-** | PA14 | 11.01 | 42.36 |
|  | PAET1 | 3.60 | 13.84 |
|  | PAET4 | 1.98 | 7.63 |
